# When Can We Safely Stop Luteal Phase Support in Fresh IVF Cycles? A Literature Review

**DOI:** 10.3389/frph.2020.610532

**Published:** 2020-12-09

**Authors:** Yossi Mizrachi, Arieh Raziel, Ariel Weissman

**Affiliations:** In vitro Fertilization Unit, Department of Obstetrics and Gynecology, The Edith Wolfson Medical Center, Holon, Affiliated With Sackler Faculty of Medicine, Tel-Aviv University, Tel-Aviv, Israel

**Keywords:** IVF, ART, luteal support, duration (time), fresh

## Abstract

There is no consensus on the optimal duration of luteal phase support (LPS) in fresh IVF cycles. Although some clinicians withdraw LPS on the day of a positive pregnancy test, most clinicians continue its administration at least up to the 8th week of gestation. In this literature review, we included several randomized clinical trials comparing early and late cessation of LPS. Most studies have found no benefit in extended administration. These studies, however, were limited by their small sample size and selection bias. Until now, only a few attempts have been made to indicate when LPS can be safely stopped based on individual patient characteristics. In conclusion, the quality and quantity of the evidence regarding LPS duration in fresh IVF cycles is currently insufficient to justify early cessation in all patients. Individualization of LPS should receive high priority in future research.

## Introduction

Luteal phase support (LPS) has a pivotal role in the establishment and maintenance of IVF pregnancies. This has been one of the earliest evidence-based subjects to become a consensus in assisted reproduction technology (ART) (1). Following ovarian stimulation and administration of human chorionic gonadotropin (hCG) for final follicular maturation, pulsatile pituitary LH secretion is severely compromised and is therefore unable to support normal function of the corpora lutea, resulting in a deficient luteal phase that must be supported pharmaceutically (2). This was initially demonstrated in GnRH agonist cycles and subsequently confirmed for GnRH antagonist cycles as well.

It has been clearly demonstrated that LPS is crucial in filling the gap between the disappearance of exogenously administered hCG for ovulation triggering and the initiation of secretion of endogenous hCG from the implanting conceptus. After implantation, embryonic hCG takes over pituitary LH in supporting the corpus luteum (CL) and maintains its function until the establishment of the luteo-placental shift, at around the 8th week of gestation (3).

Early studies have estimated that exogenously administered hCG remains in the circulation for up to 7 days (4), and that the CL has a remarkable ability to recover after a week of deprivation from gonadotropin stimulation (5). Thus, LPS in the form of exogenous progesterone and/or hCG has become an integral part of fresh IVF cycles.

In terms of duration, without clear evidence to support the administration of luteal support beyond the day of the first pregnancy test, it has become a common practice to administer LPS until the establishment of the luteo-placental shift, at 8, 10 or even 12 weeks of gestation (6).

## Review of the Literature

Several investigators have questioned the benefit of LPS administration beyond the point of pregnancy establishment. Retrospective as well as prospective studies have compared early cessation of LPS, either at the time of positive hCG test or early pregnancy ultrasound scan, to extended administration until 7–8 gestational weeks.

Kyrou et al. (7) conducted a prospective study in which 200 women with doubling in hCG levels following GnRH-antagonist cycles were randomized to early cessation of LPS, consisting of natural micronized vaginal progesterone, 16 days after embryo transfer, or extended administration up to the 7th week of pregnancy. They found similar ongoing pregnancy rates (OPRs) and abortion rates in both study groups. Goudge et al. (8) reached similar results in GnRH-antagonist cycles using intramuscular (IM) progesterone.

Other researchers demonstrated similar outcome after early and late cessation of LPS in GnRH-agonist cycles. Nyboe Andersen et al. (9) randomized 303 women undergoing GnRH-agonist cycles to receive LPS with vaginal progesterone up to the first positive pregnancy test or until the 7th week of gestation. Live birth rate (LBR) and miscarriage rate were similar. Aboulghar et al. (10) randomized 257 women undergoing GnRH-agonist cycles to receive LPS until the first ultrasound scan at 6–7 weeks or until 9–10 weeks of gestation. Miscarriage rate was similar for both study groups. In addition, several retrospective cohort studies have failed to demonstrate any benefit of extended LPS administration (11–13).

Only one early RCT showed higher clinical pregnancy rate (CPR) in women treated with a long GnRH-agonist protocol and received extended LPS consisting of IM 17α-hydroxyprogesterone caproate and 10 mg estradiol valerate (PC/EV) in an oily vehicle up to the 12th week of gestation (14).

Table 1 summarizes the characteristics of randomized clinical trials comparing short and extended LPS administration.

**Table 1:** Randomized clinical trials comparing short vs. extended luteal phase support in fresh IVF cycles.

**Study first** **author, year**	**COS protocol**	**Number of cycles**	**Dose and route of administration**	**Short LPS—timing of cessation**	**Extended LPS—timing of cessation (gestational age)**	**Results**	**Risk of selection bias**
Prietl et al. (14)	GnRH-a	120	IM P 500 mg + estradiol 10 mg tree times weekly	Day of positive pregnancy test	Week 12	Higher CPR in extended LPS group	Patients were included only if β-hCG levels increased properly
Nyboe Andersen et al. (9)	GnRH-a	303	Vaginal P 200 mg three times daily	Day of positive pregnancy test	Week 7	Comparable LBR and miscarriage rate	Patients with bleeding episodes were excluded
Aboulghar et al. (10)	GnRH-a	257	IM P 50 mg or vaginal P 600 mg daily	Week 6–7 (after US scan)	Week 9–10	Comparable miscarriage rate	Randomization occurred after US scan demonstrating heart activity.
Goudge et al. (8)	GnRH-a/antag	101	IM P 50 mg daily	Day of positive pregnancy test	Week 6	Comparable CPR, OPR, LBR	LPS continued if P level <15 ng/mL on the day of pregnancy test
Kyrou et al. (7)	GnRH-antag	200	Vaginal P 200 mg three times daily	16 days post embryo transfer	Week 7	Comparable OPR and miscarriage rate	Patients were included only if doubling in β-hCG levels occurred after 48 h
Kohls et al. (15)	GnRH-antag	220	Vaginal P 200 mg twice daily	Week 5 (after US scan)	Week 8	Comparable OPR and miscarriage rate	Patients with bleeding episodes were excluded

*COS, controlled ovarian stimulation; LPS, luteal phase support; RCT, randomized clinical trial; GnRH-a, gonadotropin-releasing hormone agonist; GnRH-antag, gonadotropin-releasing hormone antagonist; IM, intra-muscular; PR, per-rectum; P, progesterone; US, ultrasound; CPR, clinical pregnancy rate; OPR, ongoing pregnancy rate; LBR, live birth rate*.

It should be noted that in the majority of the previous studies patients were carefully selected for inclusion based on normally rising hCG levels (7, 14), absence of vaginal bleeding episodes (7, 9, 15), favorable serum progesterone levels (8, 12), serum estradiol levels (12), age (7, 15) and even normal early ultrasound scan at 5 weeks (15) or 6–7 weeks (10) of gestation. Therefore, it appears that only carefully selected, good prognosis patients were studied. In addition, previous studies were not sufficiently powered to detect small differences in LBR. Most clinicians would agree that even a small increase in LBR would justify an extended LPS administration.

In an editorial addressing one of the above-mentioned RCTs (7) George Griesinger has estimated that for a non-inferiority trial showing a difference of 4% or larger in the LBR, a sample size of 3,140 women with a positive pregnancy test would be required (16). Moreover, an optimal study should be placebo-controlled and all patients with a positive pregnancy test should be included and randomized. Specific patient populations, such as high and low responders, those with young and advanced maternal age, patients with a history of early bleeding episodes or with endometriosis and those with recurrent pregnancy loss, should be evaluated separately in detail. The choice of ovulation triggering method, hCG, GnRH agonist or their combination, should also be considered.

Despite the heterogeneity of previous studies and the bias introduced by patient selection criteria, several meta-analyses were performed to investigate the impact of extended LPS duration. In 2012, Liu et al. (17) published a meta-analysis of the above mentioned 6 randomized trials with a total of 1,201 participants. No differences were detected between patients who underwent early and late progesterone cessation in terms of LBR or OPR. A recent meta-analysis by Watters et al. (18) summarized the results of the above mentioned 6 randomized trials and one more trial by Gazvani et al. (19), which also found comparable results between short and extended LPS duration. This meta-analysis showed similar live birth, miscarriage and OPRs after early and late LPS cessation. A Cochrane review (20) found similar results. The authors of these reviews concluded that prolonged progesterone supplementation after fresh embryo transfer might be unnecessary. Finally, recent guidelines by the European Society of Human Reproduction and Embryology (ESHRE) (21) recommend that progesterone for LPS should be administered at least until the day of the pregnancy test.

While these meta-analyses provide some reassurance regarding early secession of LPS, clinicians must take into account the limitations of the included studies as mentioned above.

## The Controversy Still Exists

LPS regimens harbor discomfort and side effects and increase the cost of treatment. Therefore, clinicians should aspire to reduce the duration of LPS to the minimum. This may improve patient's convenience and compliance with treatment.

While most of the above RCTs were published more than a decade ago in the leading journals of our field, the scientific community has been very conservative and generally reluctant to adopt the practice of early cessation of LPS. This is evident in the methodology being used in recent publications from our discipline, as well as by examining the results of a series of web-based surveys (https://ivf-worldwide.com/survey.html) evaluating the practice of LPS. A close look at four such surveys conducted over the last decade (2009–2019) reveals that the majority (>60%) of clinicians world-wide administer LPS until 8 gestational weeks and beyond. This rate did not change significantly over the years.

A survey conducted in the UK showed similar results (22). Only 24% of clinics in the UK withdraw luteal support at biochemical confirmation of pregnancy. Notably, 40% of clinics reported that they continue LPS until the 12th week of gestation.

These findings represent the perception that the quality of evidence regarding early cessation of LPS is weak and insufficient to allow a change in practice. Clinicians must be confident that they comply with the principle of “primum non nocere” or “first do no harm” before introducing a routine of early LPS withdrawal.

## Individualization of Luteal Phase Support

Over the years, significant advances have been made in optimizing ovarian stimulation protocols, and individualization has become a major component of controlled ovarian stimulation (COS). The luteal phase, however, has been neglected in this sense, and the practice of “one size fits all” has become the rule in the majority of ART programs, as most patients receive the same fixed regimen of LPS.

Only a few efforts have been made to indicate when LPS can be safely stopped based on individual patient characteristics. In a retrospective cohort study, Segal et al. (12) reported the results of an early cessation of LPS based on estradiol and progesterone levels on the day of a positive pregnancy test in fresh IVF cycles. LPS was stopped in 99 patients with high serum concentrations of estradiol and progesterone (≥1,000 pmol/l and ≥110 nmol/l, respectively) on the day of a positive pregnancy test, and these were compared to 85 patients who did not meet the above criteria, in whom LPS was continued until gestational week 9. The groups did not differ with regard to the live birth and miscarriage rates. The study was limited by its retrospective design and small sample size.

Another available biochemical evidence for normal implantation and embryo development is a sufficient rise in hCG levels. In a previous RCT (7), doubling of hCG levels in 48 h was a criterion for early LPS cessation. The authors found comparable outcome in these patients compare with patients who continued LPS. Other investigators withdrew LPS only in patients with no episodes of vaginal bleeding (9, 15).

There is a crucial need to develop advanced techniques to evaluate the function of the CL starting from the mid-luteal phase as well as the efficiency of CL rescue process by the ensuing pregnancy (23). This would allow individualization of both the extent and the duration of LPS. Serum progesterone measurements can be used, but with most LPS regimens it is impossible to distinguish between ovarian and exogenously administered progesterone. Serum LH and hCG levels can be monitored as well (23). Potential solutions to this problem may include the following: (1) Administration of LPS with small doses of hCG, which is limited by the inherent risk of ovarian hyperstimulation syndrome. (2) Administration of progestins such as dydrogesterone that due to its chemical properties does not cross-react with endogenous progesterone when measured in the serum, allowing exclusive analysis of progesterone from CL origin (24). (3) Study of other yet undefined non-steroidal substances produced by the CL which may reflect its level of function.

## Conclusion

Although the current review did not include a systematic literature search, it covered the leading publications in this subject. It can be concluded that the quality and quantity of evidence regarding early cessation of LPS in fresh IVF cycles is insufficient to justify a change in the current practice of continuation until the establishment of the luteo-placental shift. Better understanding of CL function following various COS regimens and ovulation triggering agents may allow individualization of LPS regimens according to specific patient's characteristics. Individualization of LPS should receive high priority in future research.

## Author Contributions

All authors contributed substantially to the concept and design, analysis and interpretation of data, drafting, and revisions.

## Conflict of Interest

The authors declare that the research was conducted in the absence of any commercial or financial relationships that could be construed as a potential conflict of interest.
